# Nominal Versus Realized Costs of Recruiting and Retaining a National Sample of Sexual Minority Adolescents in the United States: Longitudinal Study

**DOI:** 10.2196/36764

**Published:** 2023-02-02

**Authors:** Mary Rose Mamey, Sheree M Schrager, Harmony Rhoades, Jeremy T Goldbach

**Affiliations:** 1 University of Southern California Los Angeles, CA United States; 2 Washington University St Louis St Louis, MO United States

**Keywords:** cost analysis, study recruitment, longitudinal retention, sexual minority adolescents, mobile phone

## Abstract

**Background:**

Web-based recruitment for research studies is becoming increasingly popular and necessary. When compared with the traditional methods of recruitment, these methods may enable researchers to reach more diverse participants in less time. Social media use is highly prevalent among adolescents, and the unique context of social media may be particularly important for the recruitment of sexual minority young people who would not be captured by traditional methods.

**Objective:**

This paper described the details of a national web-based study recruitment approach aimed at sexual minority adolescents across the United States, focusing on important details of this relatively novel approach, including cost, time efficiency, and retention outcomes.

**Methods:**

This study recruited sexual minority adolescents aged 14-17 years living in the United States through targeted advertisements on Facebook, Instagram, and YouTube and through respondent-driven sampling (RDS). Potential participants completed eligibility screening surveys and were automatically directed to a baseline survey if they were eligible. After baseline survey completion, additional data checks were implemented, and the remaining participants were contacted for recruitment into a longitudinal study (surveys every 6 months for 3 years).

**Results:**

Recruitment lasted 44 weeks, and 9843 participants accessed the initial screening survey, with 2732 (27.76%) meeting the eligibility criteria and completing the baseline survey. Of those, 2558 (93.63%) were determined to have provided nonfraudulent, usable study data and 1076 (39.39%) subsequently enrolled in the longitudinal study. Of the baseline sample, 79.05% (2022/2558) was recruited through Facebook and Instagram, 3.05% (78/2558) through YouTube, and 17.9% (458/2558) through RDS. The average cost of recruiting a participant into the study was US $12.98, but the recruitment cost varied by method or platform, with a realized cost of US $13 per participant on Facebook and Instagram, US $24 on YouTube, and US $10 through RDS. Participant differences (sex assigned at birth, race and ethnicity, sexual orientation, region, and urbanicity) were identified between platforms and methods both in terms of overall number of participants and cost per participant. Facebook and Instagram were the most time efficient (approximately 15 days to recruit 100 participants), whereas RDS was the least time efficient (approximately 70 days to recruit 100 participants). Participants recruited through YouTube were the most likely to be longitudinally retained, followed by Facebook and Instagram, and then RDS.

**Conclusions:**

Large differences exist in study recruitment cost and efficiency when using social media and RDS. Demographic, region, and urbanicity differences in recruitment methods highlight the need for attention to demographic diversity when planning and implementing recruitment across platforms. Finally, it is more cost-effective to retain than recruit samples, and this study provided evidence that with thorough screening and data quality practices, social media recruitment can result in diverse, highly involved study populations.

## Introduction

The use of web-based recruitment (including social media, websites, and other internet-based platforms) for research studies has become an increasingly popular tool in recent years [1,2]. Researchers have used web-based recruitment for mental health, medical, and treatment studies [3]. Web-based recruitment often surpasses traditional research recruitment strategies because it reduces costs; reaches more people in a short amount of time; and reaches more diverse populations, as social media and other web-based platforms have become a space for communities across all ages, ethnic and racial groups, gender identities, sexual orientations, and socioeconomic backgrounds [2-6].

Criticisms for web-based recruitment are not without merit. For instance, although these methods may recruit large samples in relatively short periods, they may be more or less effective with different populations. For example, Carter-Harris et al [7] found that recruiting older adults may be more difficult, as they are less likely to adopt new technology. Similarly, regardless of the breadth of sampling (be it state, national, or international), these studies are nonprobabilistic (eg, Whitaker et al [2]). Furthermore, Whitaker et al [2] found that overrepresentation of females, young adults, and individuals with higher education attainment may be common in these types of studies, although these are not unlike research as a whole, regardless of the sampling strategy used [2].

Despite some challenges, these recruitment methods may be particularly useful in certain populations, which are more difficult to reach through probability sampling approaches. In particular, an overwhelming majority of young people use social media sites. For example, Barry et al [8] found that approximately 93% of adolescents have at least one social media account and check their social media accounts at least once per day. Other studies have reported that approximately 45% of adolescents aged 13-17 years are consistently on the internet throughout the day [8], which is an increase from the 24% reported in 2015 [2]. With many young people having a Facebook or an Instagram account or actively watching and subscribing to YouTube videos [9,10], recruitment using these platforms can address the current recruitment challenges to reach young participants [1,11]. Indeed, >95% of adolescents have a smartphone, and many have a Facebook (51%) or an Instagram (15%) account [8]. As many adolescents spend their time in these web-based spaces, targeted advertisements through social media platforms can make this population more accessible [12], and research has shown success in recruiting on these platforms [13-15].

A unique feature of web-based recruitment, particularly through social media platforms, is the ability to access hard-to-reach samples. In particular, recruitment and retention of sexual minority adolescents may be especially challenging because of stigma or discrimination based on their sexual orientation [11,13,16,17]. Because of discrimination and bias, many people—particularly young people—may not be safe or feel comfortable disclosing their sexual orientation to their families, communities, or schools. As such, conducting research recruitment that targets *visible* or disclosed identity categories will necessarily exclude a large swath of sexual minority adolescents. Although researchers have developed creative strategies for the recruitment of this population in the past (eg, full-school samples with anonymous data and community centers [12,18,19]), this younger population presents a host of difficulties for contact. Adolescents may not yet be “out” in any of these spaces [11], they may not engage in centers or school clubs for fear of ostracism [20] or lack of availability, or they may live in areas without access to these types of resources (eg, gender-sexuality alliances and community centers). Conducting recruitment at the school level may be one way to capture this population, as long as the study provides adequate and convincing confidentiality protections for adolescents who have not widely disclosed their identity. However, such samples are expensive to gather and also require surveying a large percentage of adolescents who will not be part of the target sample (as research in large-scale school samples estimate that 8%-10% of adolescents identify as a sexual minority) [21]. Furthermore, for researchers interested in the social determinants of health (eg, racism and heterosexism), national studies of adolescents such as Monitoring the Future [22] and the Youth Risk Behavior Surveillance Survey [23] understandably do not assess these more nuanced experiences.

Another recruitment method to access hard-to-reach populations is respondent-driven sampling (RDS; [24-26]). RDS allows for snowball-like recruitment; a study participant is asked to recruit others like them and in turn usually receives an incentive for each successfully recruited participant they refer. This type of recruitment strategy has been very successful with hidden populations, including men who have sex with men [27], people with HIV [28], and transgender and substance-using populations [29]. However, the benefits of coupling RDS with social media recruitment remain unknown.

Given the growing evidence supporting the utility of the web-based recruitment of research participants, known difficulties in the existing methods for recruiting sexual minority adolescents given their sometimes-hidden status, and the fact that most adolescents use social media and other web-based platforms, this paper outlines the recruitment process and lessons learned in a large-scale, longitudinal study of sexual minority adolescents. This paper aimed to describe in detail our web-based recruitment approach and present quantitative data on cost and retention outcomes, including the cost of all types of recruitment, whether direct web-based (via targeted advertising) or RDS recruitment is more cost-efficient, and which platform (Facebook and Instagram vs YouTube) is more cost-efficient. We also present our findings on the “true” cost of web-based and RDS recruitment and retention in a national sample of hard-to-reach adolescents over 2 years, which method was most successful at retention, and whether demographic characteristics affected any of these processes.

## Methods

### Ethics Approval

Ethics review and approval was obtained by the The Washington University in St Louis Institutional Review Board (IRB ID: 202212035).

### Overview

This study recruited adolescents aged 14 to 17 years in the United States who identified as nonheterosexual and cisgender male or female through targeted advertisements on Facebook, Instagram, and YouTube channels and through RDS. Recruitment for baseline participation lasted 44 weeks between May 2018 and April 2019. Facebook and Instagram advertisements went live on May 15, 2018, and YouTube advertisements went live on January 9, 2019. RDS was available to all the participants and ran for the entire duration of 44 weeks that the survey was open.

### Social Media

#### Facebook and Instagram Targeting

Facebook’s Ads Manager (which delivers advertisements for both Facebook and Instagram) allows targeting by users’ demographics (eg, age, gender, and location) and interests (eg, activities or sports [5,9,10]). In this study, we used demographic targeting for age (14-17 years), sex (girls and boys), and location (geographic location or region and urbanicity). To balance the recruitment of different participants in our study, we used a strategy that accounted for the combinations of these characteristics; the 5 geographic locations (West, Southwest, Midwest, Northeast, and Southeast) each contained 4 groups: urban girls, rural girls, urban boys, and rural boys. This resulted in 20 targeted advertisements. Facebook allowed bulk uploading of zip codes for target locations (ie, region and urbanicity) but only up to 2500 per advertisement. Multiple advertisements were created for groups with >2500 zip codes (eg, urban girls in the West), resulting in 44 targeted advertisements being disseminated. Specific advertisements were closed when the sample became saturated with a single demographic characteristic. This allowed for the distribution among certain characteristics to maintain better balance. Advertisements were also targeted to those categorized by Facebook as having “interest group” affiliations related to the LGBTQ (lesbian, gay, bisexual, transgender, and queer) community by using keywords for interests such as “gay-friendly,” “homosexuality,” and “LGBT community.” Facebook and Instagram share an advertising platform, so advertisements posted via Facebook were autopopulated on Instagram.

#### YouTube Targeting

After starting Facebook and Instagram recruitment, our youth advisory group indicated that their peers used YouTube as another common source of information. We started expanding our recruitment to YouTube by using keywords such as “LGBTQ,” “gay,” and “coming out” to identify YouTube channels relevant to the target population. Channels with the highest subscriber counts were further evaluated to identify large visibility, reach, and engagement (operationalized as the number of video views for each channel’s top 3 most-viewed videos). Of the 47 possible YouTube channels identified as having high visibility and engagement among LGBTQ adolescents, 23 (49%) were available for advertising and were confirmed as relevant to sexual minority adolescents per discussions with youth advisors. The Google advertising system was used to place these advertisements on the YouTube channel pages identified.

#### Advertisements

Similar advertisements were used on both Facebook and Instagram and YouTube; the advertisement text asked those who saw the advertisement to “Share Your Voice!” Information about the study was also included: “Be on the forefront of LGBTQ research and earn up to $195! We’re doing a research study to learn about the experience of growing up as a lesbian, gay, bisexual, or pansexual young person. If you’re eligible, you’ll receive a $15 gift card for taking the first survey, and some people will be invited to be part of future studies with additional gift cards. Click here to start.” Each advertisement was accompanied by 2 images. The first image in all the advertisements was a city skyline with a rainbow flag and the words “Share Your Voice,” and the second image was tailored to boys or girls, depicting either a young man or a young woman “shouting” the “Share Your Voice” tagline.

### Recruitment via RDS

A participant who enrolled in the study had the opportunity to recruit up to 3 people like them and received a monetary incentive of a US $10 gift card for each successful recruit. Those who confirmed their interest in recruiting others received an email that contained 3 unique links, each to be sent to only one referral. A prompt offered language that the participant could use to send the links to their friends.

### Survey Completion

Potential participants who clicked an advertisement from one of the social media platforms or were recruited through RDS were routed to a Qualtrics (Qualtrics International Inc) survey page. Screener questions were asked to determine eligibility. Eligible participants were aged between 14 and 17 years, identified as the same gender assigned at birth (ie, cisgender), and were currently living in the United States. Adolescents also had to identify as nonheterosexual based in an item assessing sexual orientation (ie, identify as “Mostly heterosexual [straight], but somewhat attracted to people of your own gender”; “Bisexual or pansexual”; “Mostly homosexual [gay/lesbian] but somewhat attracted to people of the opposite gender”; “100% homosexual [gay/lesbian]”; or “Unsure”). These eligibility requirements were set following similar guidelines used in the development and validation of the Sexual Minority Adolescent Stress Inventory (SMASI) [30,31]. The SMASI is a measure used to assess the stress experienced by sexual minority adolescents above and beyond general adolescent stress and was initially developed and validated using a cisgender sample. Because a main focus of the study was to longitudinally validate the SMASI (eg, assessing the longitudinal invariance), it was necessary to focus enrollment on youths who matched the eligibility criteria of the study in which the original measure was developed [30]. Those who were not eligible for participation were thanked for their interest in the study and had the opportunity to fill out information to be contacted for future studies. Those who were eligible for participation reviewed the institutional review board–approved assent form and were asked whether they assented. Those who did not assent were thanked for their time, whereas those who assented accessed the rest of the survey. After completing the survey, the participants were routed to a payment survey to enter their email address for compensation. They were also asked 2 questions pertaining to RDS—specifically, whether they knew other sexual minority adolescents and whether they would be willing to recruit them into the study. They were also asked whether they would be interested in being a part of a longitudinal study and, if so, to provide information on how best to reach them (eg, email, phone, or text).

Rigorous steps were taken to identify any fraud (eg, duplicate participants and participants who failed the screener and reaccessed the survey with different answers) and poor-quality data (low validation or attention scores and low survey duration). Of the 9843 participants who initially accessed the survey, 2732 (27.76%) met the eligibility criteria for inclusion in the study. Steps were taken to ensure the best-quality data. Participants were denied access to the full survey if they were ineligible based on screener questions, closed the survey before the end of the screener, or did not assent to participate. Postsurvey data-cleaning efforts resulted in the removal of participants who closed the survey before its completion, made multiple attempts to take the survey, were not in the payment survey, or accessed the survey through a fraudulent channel. Further cleaning was then conducted to exclude those with fraudulent or extremely poor-quality data, such as unrealistically short survey duration, low validation or attention control scores (eg, “Please select the response option ‘None of the above’” answered incorrectly), successful repeat survey completions by the same individual, or missing data on key outcome measures [32]. After the completion of these steps, 93.63% (2558/2732) of respondents provided usable data in the baseline survey.

### Longitudinal Recruitment and Retention

Participants who were eligible for the baseline study and indicated an interest in participating in the longitudinal study were enrolled for follow-up surveys (1076/2732, 39.39%). Participants were contacted every 6 months based on the date of the baseline survey. At each survey, participants assented or consented to the survey and received gift cards that increased by US $5 at each wave. To keep participants engaged during the study and ensure that contact information stayed up-to-date, check-in surveys were sent every month. A single question determined whether their contact information had changed, and participants had the opportunity to update it. Respondents who participated in the check-ins were entered into a raffle to win US $100 each time they completed the monthly survey.

## Results

### Recruitment Cost

Of the 2558 participants who were successfully enrolled and retained in the initial study, 2022 (79.05%) were recruited through Facebook and Instagram, 78 (3.05%) were recruited through YouTube, and 458 (17.9%) were recruited through RDS. Table 1 provides the total cost for all phases of the study.

Advertisements on Facebook and Instagram remained active during the 44 weeks of the study and cost US $26,571.62. The survey was advertised 3,035,126 times and reached the profiles of 617,927 unique people, of whom 18,469 (2.99%) clicked the advertisement to access the survey. This resulted in a nominal cost of US $1.44 per click. Of these 18,469 individuals, 7773 (42.09%) individuals accessed the survey, which began with screening questions after clicking the advertisement. Steps were taken to ensure the best-quality data. Participants were denied access to the full survey if they were ineligible based on screener questions, closed the survey before the end of the screener, or did not assent to participate. Data from 121 Facebook and Instagram participants were excluded for these reasons, resulting in 2022 Facebook and Instagram participants—representing 10.9% of all advertisement clicks (n=18,469) on these platforms—who successfully completed the study, bringing the realized (adjusted) cost of Facebook and Instagram advertising to US $13.14 per participant.

In comparison, advertisements on YouTube were only active for 3 weeks of the study for a total cost of US $1892.89. Of 1621 clicks, most came from a single channel (n=960, 59.22%); they yielded a nominal cost of US $1.16 per person. Of the 1621 clicks, 538 (33.19%) individuals accessed the survey. However, after data screening and cleaning efforts similar to those previously described, only 78 (4.81%) eligible participants completed the survey after recruitment through YouTube advertisements, resulting in a realized cost of US $24.27 per participant. James Charles, a beauty vlogger and makeup artist, brought in the most eligible participants for a single channel (29/78, 37%).

Finally, participants who shared RDS referral links brought in 473 additional survey responses and received US $10 for their eligible referrals. Unlike direct advertising, in which the cost of the advertisement is incurred before the potential participant accesses the survey, RDS referrals only triggered referral payments after the referred participant successfully screened as eligible and completed the survey. Thus, the adjusted cost of the 458 (96.83%) out of 473 participants whose data were retained for the study was US $10.33. Overall, the average adjusted cost of recruiting a single participant into the study was US $12.98, with RDS being the most cost-efficient method.

In addition to the costs associated with attracting potential participants to the study, participants received US $15 for completing the baseline survey. The total payout was US $40,980 to 2732 participants who were initially screened as eligible for study participation based on the study inclusion criteria. After removing data from participants whose efforts were determined to be fraudulent or of extremely poor quality as described previously, 2558 participants were retained in the baseline sample, resulting in a realized cost per retained baseline survey of US $16.02. Combined with the associated recruitment costs, this resulted in a total realized cost of US $29.00 per baseline study participant.

**Table 1:** Costs associated with recruitment, participation, and retention by initial recruitment method.

Phase	Information	Total cost (US $)	Cost description	Unique participants paid, n	Participants retained, n (%)^a^	Nominal cost per participant (US $)	Realized (adjusted) cost per participant (US $)	Cost difference (US $)
Advertising recruitment	Facebook and Instagram	26,571.62	18,469 clicks	2143	2022 (10.9^b^)	1.44	13.14	11.69
Advertising recruitment	YouTube	1892.89	1621 clicks	81	78 (4.8^c^)	1.16	24.27	23.11
RDS^d^ recruitment	Referrals	4730	473 referrers at US $10 per referral	508	458 (90.2^e^)	10.00	10.33	0.33
Study enrollment	Baseline	40,980	US $15 baseline survey incentive	2732	2558 (93.6)	15.00	16.02	1.02
Retention	6 months	19,940	US $20 6-month survey incentive	997	969 (97.2)	20.00	20.58	0.56
Retention	12 months	24,025	US $25 12-month survey incentive	961	945 (98.3)	25.00	25.42	0.40
Retention	18 months	27,570	US $30 18-month survey incentive	919	912 (99.2)	30.00	30.23	0.20
Retention	24 months	31,430	US $35 24-month survey incentive	898	894 (99.6)	35.00	35.16	0.12
Retention	Raffles	3100	US $100 monthly raffle prize	31	31 (100)	100.00	100.00	0.00

^a^The denominator of each percentage is the number of unique participants paid in the same row, except for those that indicate otherwise.

^b^10.9% of attempts out of 18,469 clicks.

^c^4.8% of attempts out of 1621 clicks.

^d^RDS: respondent-driven sampling.

^e^90.2% of referrals out of 473 referrers at US $10 per referral.

### Demographic Differences in Recruitment

The large cost discrepancies that existed across these methods may also shed light on which avenues may be more successful at accessing young people with specific demographic characteristics. Because Facebook and Instagram drew most participants who were eligible for the study, few discrepancies were observed in that group compared with the overall sample. However, both YouTube and RDS provided insight into the categories of people who may be more inclined to access the survey through these methods. Table 2 summarizes the demographic information for the overall sample and by recruitment method. The results of the chi-square test for each group were significant at *P*<.001.

**Table 2:** Demographics of participating youths (N=2558).

Demographics			Total, n (%)		FB^a^ and IG^b^ (n=2022), n (%)		YouTube (n=78), n (%)		RDS^c^ (n=458), n (%)		Chi-square (*df*)			*P* value	
**Sex assigned at birth**												44.2 (1)			<.001
	Male	912 (35.65)		779 (38.53)		7 (8.97)		126 (27.51)							
	Female	1646 (64.35)		1243 (61.47)		71 (91.03)		332 (72.49)							
**Age (years)**												28.4 (3)			<.001
	14	260 (10.16)		198 (9.79)		16 (20.51)		46 (10.04)							
	15	568 (22.2)		425 (21.02)		26 (33.33)		117 (25.55)							
	16	888 (34.71)		700 (34.62)		19 (24.36)		169 (36.9)							
	17	842 (32.92)		699 (34.57)		17 (21.79)		126 (27.51)							
**Race and ethnicity**												43.1 (5)			<.001
	Native American, American Indian, or Alaska Native	59 (2.31)		50 (2.47)		0 (0)		9 (1.97)							
	Asian or Pacific Islander	164 (6.41)		120 (5.93)		7 (8.97)		37 (8.08)							
	Black or African American	199 (7.78)		151 (7.47)		1 (1.28)		47 (10.26)							
	White	1548 (60.52)		1269 (62.76)		51 (65.38)		228 (49.78)							
	Latino or Hispanic	370 (14.46)		261 (12.91)		11 (14.1)		98 (21.4)							
	Multiracial	217 (8.48)		170 (8.41)		8 (10.26)		39 (8.52)							
**Sexual attraction**												39.3 (4)			<.001
	Mostly heterosexual	137 (5.36)		92 (4.55)		2 (2.56)		43 (9.39)							
	Bisexual or pansexual	1035 (40.46)		785 (38.82)		42 (53.85)		208 (45.41)							
	Mostly homosexual	491 (19.19)		400 (19.78)		17 (21.79)		74 (16.16)							
	100% homosexual	850 (33.23)		710 (35.11)		16 (20.51)		124 (27.07)							
	Unsure	45 (1.76)		35 (1.73)		1 (1.28)		9 (1.97)							
**Sexual identity**												44.9 (4)			<.001
	Gay	618 (24.16)		541 (26.76)		6 (7.69)		71 (15.5)							
	Lesbian	440 (17.2)		329 (16.27)		19 (24.36)		92 (20.09)							
	Bisexual or pansexual	1166 (45.58)		887 (43.87)		42 (53.85)		237 (51.75)							
	Queer	85 (3.32)		72 (3.56)		4 (5.13)		9 (1.97)							
	Other	249 (9.73)		193 (9.54)		7 (8.97)		49 (10.7)							
**Region**												34.9 (4)			<.001
	West	630 (24.63)		465 (23)		32 (41.03)		133 (29.04)							
	Southwest	336 (13.14)		281 (13.9)		8 (10.26)		47 (10.26)							
	Midwest	448 (17.51)		380 (18.79)		14 (17.95)		54 (11.79)							
	Southeast	592 (23.14)		465 (23)		11 (14.1)		116 (25.33)							
	Northeast	552 (21.58)		431 (21.32)		13 (16.67)		108 (23.58)							
**Urbanicity**												33.9 (1)			<.001
	Rural	504 (19.7)		446 (22.06)		12 (15.38)		46 (10.04)							
	Urban	2054 (80.3)		1576 (77.94)		66 (84.62)		412 (89.96)							

^a^FB: Facebook.

^b^IG: Instagram.

^c^RDS: respondent-driven sampling.

Most participants were female (1646/2558, 64.35%), with similar distributions for both Facebook and Instagram (1243/2022, 61.47%) and RDS (332/458, 72.5%). However, 91% (71/78) of the participants recruited via YouTube were female (*χ*^2^_1_=44.2, *P*<.001). A greater proportion of adolescents aged 14 years (16/78, 20%) and 15 years (26/78, 33%) came in through YouTube compared with Facebook and Instagram (198/2022, 9.79% and 425/2022, 21.02%, respectively) and RDS (46/458, 10% and 117/458, 25.6%, respectively; *χ*^2^_3_=28.4, *P*<.001). Race also differed across recruitment methods (*χ*^2^_5_=43.1, *P*<.001). Most notably, the sample was primarily composed of White participants (1548/2558, 60.52%), with Latino and Hispanic participants making up the second largest represented group at only 14.46% (370/2558). However, this is reflective of the country’s current race or ethnicity [33]. YouTube (7/78, 9%) and RDS (37/458, 8.1%) had a larger proportion of Asian and Pacific Islander participants than Facebook and Instagram (120/2022, 5.93%); a disproportionally lower Black and African American group came in through YouTube (1/78, 1%) compared with Facebook and Instagram (151/2022, 7.47%) and RDS (47/458, 10.3%); fewer White participants came in through RDS (228/458, 49.8%) compared with Facebook and Instagram (1269/2022, 62.76%) and YouTube (51/78, 65%), and more Latino and Hispanic participants came in through RDS (98/458, 21.4%) compared with Facebook and Instagram (261/2022, 12.91%) and YouTube (11/78, 14%).

Sexual identity also showed differences in recruitment methods (*χ*^2^_4_=39.3, *P*<.001). RDS had higher rates of participants who identified as mostly heterosexual (43/458, 9.4%) than both Facebook and Instagram (92/2022, 4.55%) and YouTube (2/78, 2%) and lower rates of those who identified as mostly homosexual (74/458, 16.2%) than Facebook and Instagram (400/2022, 19.78%) and YouTube (17/78, 22%). YouTube had higher rates of participants who identified as bisexual or pansexual (42/78, 54%) compared with Facebook and Instagram (785/2022, 38.82%) and RDS (208/458, 45.4%). YouTube had a lower rate of participants who identified as 100% homosexual (16/78, 20%) than both Facebook and Instagram (710/2022, 35.11%) and RDS (124/458, 27.1%). Sexual orientation also showed differences among recruitment methods (*χ*^2^_4_=44.9, *P*<.001). YouTube had a lower proportion of participants who identified as gay (6/78, 8%) compared with Facebook and Instagram (541/2022, 26.76%) and RDS (71/458, 15.5%) but a higher proportion of participants who identified as lesbian (19/78, 24%) compared with Facebook and Instagram (329/2022, 16.27%) and RDS (92/458, 20.1%).

Differences in region (*χ*^2^_4_=34.9, *P*<.001) showed that YouTube had a greater proportion of those who lived in the West (32/78, 41%) and a lower proportion of those who lived in the Southeast (11/78, 14%) than Facebook and Instagram (465/2022, 23% and 465/2022, 23%, respectively) and RDS (133/458, 29% and 116/458, 25.3%, respectively). Differences in urbanicity were found between recruitment methods (*χ*^2^_1_=33.9, *P*<.001), with a greater proportion of rural participants coming in through Facebook (446/2022, 22.06%) compared with YouTube (12/78, 15%) and RDS (46/458, 10%). Although there was a disproportionate number of urban (2054/2558, 80.3%) versus rural (504/2558, 19.7%) participants, this was reflective of the country’s percentages.

### Cost of Facebook and Instagram Advertisements

The Facebook Ads Manager provided more detailed information on the breakdown of cost per targeted group. Advertisements targeted 3 characteristics for equal distribution, gender, region, and urbanicity, resulting in the 20 unique groups described previously. Facebook allowed bulk uploading of zip codes for target locations (ie, region and urbanicity), but only up to 2500 zip codes were allowed per advertisement. Multiple advertisements were created for groups with > 2500 zip codes (eg, urban girls in the West), resulting in 44 targeted advertisements. All 44 advertisements could be advertised on a single day, resulting in 1286 “ad days.” Table 3 shows the breakdown of the advertisements per group.

**Table 3:** Facebook and Instagram advertising cost by target group^a^.

			Total cost (US $)		Number of days open		Number of reaches		Cost per reach (US $)		Cost for recruitment (US $)
**Gender**											
	Male	15,043.61		724		237,384		0.06		16.48	
	Female	11,528.01		562		380,543		0.03		7.00	
**Region**											
	West	4663.43		189		101,435		0.05		7.39	
	Southwest	5468.43		123		139,590		0.04		16.28	
	Midwest	6062.16		238		139,880		0.04		13.53	
	Southeast	5643.79		396		126,643		0.04		9.53	
	Northeast	4733.81		340		110,379		0.04		8.57	
**Urbanicity**											
	Rural	14,588.29		625		200,385		0.07		28.89	
	Urban	11,983.33		661		417,542		0.03		5.83	

^a^Facebook Ads Manager allows targeting by zip code and gender but not by other demographic characteristics.

Advertisements targeting girls cost US $11,528.01 and spanned 562 ad days, resulting in a cost of US $20.51 for each day the advertisement was visible. These advertisements were seen by 380,543 unique girls, costing US $0.03 per view. With the successful recruitment of 1646 female participants into the study, this group cost an average of US $7.00 (SD $2.33) per participant in advertising to recruit. Advertisements targeting boys cost more, at US $15,043.61, but resulted in a daily cost of US $20.78, which is similar to that of the advertisements targeting girls because the advertisements targeting boys were open for a longer period (724 days). These advertisements reached 237,384 unique male participants and resulted in a cost of US $0.06 per view, which is twice that of the advertisements for female participants. Similarly, with 913 boys enrolled in the study, successfully recruiting a male participant through an advertisement on Facebook and Instagram cost US $16.48, more than twice as much as the cost for successfully recruiting a female participant on these platforms. The recruitment of male participants through Facebook and Instagram both took longer and cost more than that of their female counterparts (*z*=68.48; *P*<.001).

The cost of advertisements significantly differed across regions (all comparisons: *P*<.001), with advertisements for those living in the West costing the least (US $4663.43) and for those in the Midwest costing the most (US $6062.16). The length of time required to recruit a sufficient sample also differed by region; those in the Southwest were recruited fastest (123 ad days), whereas those in the Southeast took >3 times longer to recruit (396 ad days). All regions cost a comparable US $0.04 or US $0.05 per view, suggesting differences in recruitment efficiency by region. Overall, it was much less expensive to advertise to and successfully recruit participants from the West (US $7.39), Northeast (US $8.56), and Southeast (US $9.53) than those living in the Midwest (US $13.53) and Southeast (US $16.28). Total advertising costs for those living in urban (US $11,983.33) and rural (US $14,588.29) areas also showed large differences in both cost per view and overall cost of recruitment, although ad days were similar (rural: 625 ad days; urban: 661 ad days). Advertisements for those in urban settings cost US $0.03 per view and US $5.83 for successful recruitment. However, advertisements for those in rural settings cost US $0.07 per view and US $28.89 for successful recruitment, >5 times the cost of advertisements for their urban counterparts (*z*=98.11; *P*<.001).

### Overall Cost

The overall cost by recruitment methods was calculated as the realized cost of advertising or referral plus the adjusted per-participant cost of the baseline survey. Advertisements on Facebook and Instagram resulted in a total cost of US $29.15 per participant, those on YouTube resulted in a total cost of US $40.28 per participant, and RDS resulted in a total cost of US $26.31 per participant. RDS was a significantly more cost-efficient recruitment method compared with Facebook and Instagram (*z*=10.56; *P*<.001) and YouTube (*z*=18.39; *P*<.001). However, Facebook and Instagram proved to be more time efficient, with an average of 45 participants recruited per week, compared with RDS (approximately 10 participants per week) and YouTube (approximately 26 participants per week).

To better understand and compare the cost, time, and participant exclusion rates by recruitment method, the values were standardized to 100 participants (Table 4). For every 100 participants, the cost of recruitment was US $1314 for Facebook and Instagram, US $2427 for YouTube, and US $1033 for RDS, confirming that RDS was the most cost-efficient approach for this sample. To successfully recruit 100 participants into the study, it would take approximately 15 days of Facebook and Instagram advertising, 27 days of YouTube advertising, and 70 days of RDS referrals, suggesting that Facebook and Instagram advertisements were the most time-efficient option. Finally, the rate at which participants successfully provided usable data to the study was also examined. For every 100 participants who received the US $15 incentive for completing the baseline study, we would expect to exclude data from 5.6 Facebook and Instagram users, 3.7 YouTube users, and 9.6 RDS referrals, suggesting that YouTube was the most efficient method with regard to data quality.

**Table 4:** Comparison of expected cost, time, and exclusion rate per 100 participants by recruitment method.

Recruitment method	Cost (US $)	Time to recruit (days)	Excluded participants (per 100)
Facebook and Instagram	1314	15.22	5.6
YouTube	2427	26.95	3.7
RDS^a^	1031	69.93	9.6

^a^RDS: respondent-driven sampling.

### Retention Cost

**Figure 1:**
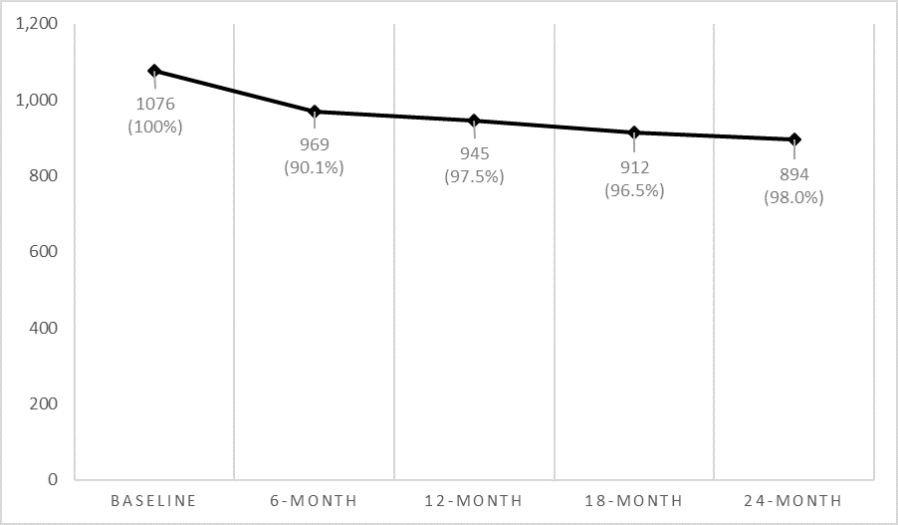
Participation completion.

Of the 2558 participants with retained baseline survey data, 2073 (81.04%) indicated an interest in being contacted for additional research opportunities and were offered enrollment in the longitudinal portion of the study. Among these 2073 participants, 934 (45.06%) were lost to contact, 49 (2.36%) declined participation, and 14 (0.68%) were excluded from follow-up for other reasons (eg, not being able to remember the study password that ensured that the research staff were contacting the right participant). Thus, 51.91% (1076/2073) of participants were successfully recruited into the longitudinal portion of the study. A total of 5 waves of data and 4 follow-up surveys occurred during the first 2 years of data collection, with follow-ups gathered at 6 months, 12 months, 18 months, and 24 months. Figure 1 shows a survival curve of retention during the first 2 years of the study, which includes the total number of participants with complete data and the percentage of retention by wave. Although the largest decrease in participant completion occurred between the baseline and 6-month surveys (969/1076, 90.1%), the retention rates remained extremely high between 6 and 12 months (945/969, 97.5%), 12 and 18 months (912/945, 96.5%), and 18 and 24 months (894/912, 98%). Overall, 75.7% (812/1072) of the participants completed all 5 waves of data.

Table 1 shows the cost breakdown of the retention of these participants at each wave. Each completed survey followed an escalating payment structure with an increase by US $5: US $20 at 6 months, US $25 at 12 months, US $30 at 18 months, and US $35 at 24 months. Because the longitudinally enrolled participants had been thoroughly vetted for fraudulent or poor-quality data before being asked to participate in the larger study, the nominal costs and real costs were very similar, as the exclusion of participants’ data was rare. At the 6-month wave, US $19,940 was paid to 997 participants at the corresponding rate of US $20. Of these 997 participants, 969 (97.2%) participants were retained for analysis, putting the real cost at US $20.58 (US $0.58 difference). The gap between nominal and real costs successively narrowed at the 12-month (US $0.42), 18-month (US $0.23), and 24-month (US $0.16) follow-ups.

To keep longitudinally enrolled participants engaged during the study, monthly requests to provide updated contact information were sent to all participants with an associated raffle drawing. The cost of each monthly raffle is included in Table 1; to date, there have been 31 months of US $100 payouts to raffle winners, totaling US $3100.

### Social Media Versus RDS

Of the 1076 participants who were longitudinally enrolled in the study, 874 (81.23%) came in through Facebook and Instagram, 45 (4.18%) came in through YouTube, and 157 (14.59%) came in through RDS. These findings are similar to those related to the recruitment of the 2558 participants enrolled in the baseline survey. However, those who were longitudinally enrolled and those who were not longitudinally enrolled had significant differences. Those recruited through YouTube were more likely to be retained longitudinally than those in the Facebook and Instagram group (odds ratio [OR] 1.79, 95% CI 1.45-2.21; *P*=.01) and the RDS group (OR 2.62, 95% CI 1.61-4.28; *P*<.001). Those who came in through RDS had much lower odds of being retained longitudinally than those in the Facebook and Instagram group (OR 0.68; 95% CI 0.39-1.02; *P*<.001). As noted previously, although advertisement on YouTube was the most expensive method in terms of recruiting and enrolling participants for the baseline survey, it was the most efficient method for retaining participants for longitudinal enrollment.

### Longitudinal Differences

Relatively minor differences existed among the longitudinally enrolled participants across demographics, demonstrating success in keeping a diverse group of participants over time. By 24 months, 86.8% (624/719) of the female participants remained in the study compared with 76.2% (272/357) of the male participants (*χ*^2^_1_=19.2, *P*<.001). Those who identified at baseline as lesbian (157/183, 85.8%), bisexual or pansexual (471/546, 86.3%), and queer (32/39, 82%) all had similar retention rates, with slightly lower retention rates among those who identified as gay (180/237, 75.9%) or another sexual orientation (56/71, 79%; *χ*^2^_4_=14.5, *P*<.001). Retention rates were similar between the racial and ethnic groups: White (530/625, 84.8%), Asian and Pacific Islander (60/72, 83%), multiracial (92/111, 82.9%), Black and African American (72/90, 80%), Latino and Hispanic (118/147, 80.3%), and Native American (24/31, 77%). Retention was equally comparable across age cohorts at baseline: 14 years old (97/115, 84.3%), 15 years old (214/264, 81.1%), 16 years old (304/362, 84%), and 17 years old (280/334, 83.8%), suggesting that age did not play a role in retention. Those who lived in rural (175/213, 82.1%) and urban (721/863, 83.5%) areas had similar retention rates, and 2-year retention did not vary substantially by US region (Southwest: 130/149, 87.2%; Midwest: 159/188, 84.6%; Northeast: 177/213, 83.1%; West: 229/278, 82.4%; and Southeast: 201/248, 81%). There were no significant differences in 2-year retention by race and ethnicity, age cohort, urbanicity, or region.

## Discussion

### Principal Findings

A national study with 2 years of follow-up data was used to examine the true cost of recruitment and retention of sexual minority adolescents. Participants were recruited through Facebook and Instagram advertisements, YouTube advertisements, and RDS referrals not only to access this hard-to-reach population but also to understand which method may be the most cost-efficient, time efficient, and successful in recruiting participants for the longitudinal portion of the study. Although the nominal cost of advertising based on the number of individuals approached to participate in the study was only US $2.97, after accounting for ineligible youths who viewed and interacted with advertisements, fraudulent attempts at study participation, and the exclusion of low-quality data, the realized cost of attracting an eligible person to the study averaged to US $12.97. It should be noted that a challenge in comparing our results to other published studies (including systematic reviews; eg, Whitaker et al [2]) is the inclusion criteria across studies. Given the narrow focus of our recruitment efforts (eg, cisgender sexual minority adolescents aged 14-17 years), we expected our costs to be higher than those of general US population–based recruitment.

Large expenses and discrepancies between nominal and realized costs existed in the recruitment process; RDS was the most cost-efficient method, and RDS and Facebook and Instagram advertising were both more cost-efficient than YouTube advertising. However, Facebook and Instagram were much more time efficient than RDS, bringing in 79.05% (2022/2558) of the study participants compared with 17.9% (458/2558) via RDS during the 44 weeks. It is perhaps not surprising that RDS was the most effective approach, as it relies upon peer referral and is, therefore, a more trusted source. Facebook and Instagram (largely driven by Instagram) likely come next because (1) youths in our target demographic are heavily represented on Instagram and (2) the algorithms used by Facebook and Instagram to target potential participants are highly effective in ensuring that the appropriate audience has access to study recruitment materials. As YouTube advertising focuses on channels, we relied largely upon input from youths in the target demographic based on which channels they most frequently watched. In the absence of a well-constructed algorithm of users (which YouTube has less of, given that no log-in is required to view most videos), this meant that our advertisement was not necessarily being seen by only the target audience and was, therefore, resulting in more clicks that had, however, far less specificity.

Demographics played a large role in both the type and cost of recruitment. Each characteristic grouping contributed to statistically significant differences in type of recruitment: gender, age, sexual attraction, sexual orientation, race and ethnicity, region, and urbanicity. Furthermore, differences in the cost of recruitment based on gender, region, and urbanicity were observed for those recruited through Facebook and Instagram. Our findings are similar to those of the systematic review by Whitaker et al [2], where they found that overrepresentation of females, young adults, and individuals with higher education attainment may be common in these types of studies. However, it should also be noted that our sample was fairly representative in comparison with the Census (2021; [34]) on race, ethnicity, and urbanicity. For example, our sample was composed of approximately 58.1% (625/1076) non-Hispanic White participants, whereas the most recent census found that 76% of the individuals identify as “White only.”

Nominal (US $15) and real (US $16.01) costs for the completion of the baseline survey showed small differences attributable to data exclusion after payment, and each of the 4 successive waves lessened the gap between these 2 costs. Although no cost differences existed in the longitudinal portion of the sample, as everyone received the same amount at each completed wave, differences were observed in the initial recruitment method in longitudinal enrollment and retention. Those recruited through RDS were surprisingly less likely to enroll longitudinally, whereas those recruited through YouTube were the most likely to enroll longitudinally. Although it is difficult for us to understand the exact reason for this change, future research should ask participants how the platform may influence their behaviors in enrolling and maintaining participation in research studies. Demographic differences were not found to vary in meaningful ways, suggesting that these strategies may be used to multiply the number of marginalized youths participating in research studies (eg, racial and ethnic minority and rural youths).

Importantly, despite the large differences between nominal and actual costs of recruitment, data-screening practices are necessary to ensure excellent-quality data with large internet samples [35-39]. The initial investment of recruiting a trustworthy sample has shown added value over time, with the wave-over-wave retention rates all exceeding 90% and the 2-year study retention exceeding 80% in nearly all demographic subgroups. The total cost per participant who completed all waves was US $140.26 versus the nominal cost of US $126.61, with most of the difference arising from the costs associated with recruitment. We expected that those recruited through RDS would be more inclined to participate in the longitudinal portion of the study because having a friend involved in the study would be mutually reinforcing. However, recruitment through social media channels, particularly YouTube, proved to be more successful in enrolling participants longitudinally.

It should be noted that the advertising recruitment methods outlined in this study reflect the capacities of advertising platforms available for Facebook and Instagram and YouTube at the time of our recruitment activities (May 2018 to April 2019). Given the growing concerns about privacy and disinformation on social media, changes over time in these advertising platforms are inevitable, particularly regarding the ability to focus on vulnerable or marginalized populations. Relevant to our findings in this paper, for example, in summer 2021, Facebook’s advertising platform discontinued the use of interest group targeting for anyone younger than 18 years [40], and starting in 2022, all interest group targeting related to sexual orientation, health, political beliefs, or religion was banned [41]. As user interactions continue to shift in this space, advertising methods will need to continue to evolve. In light of these changes in Facebook’s platform, future recruitment efforts, such as those outlined in this paper, will involve broader (and, therefore, more costly) advertising efforts, likely resulting in more ineligible participants during screening and a lower ratio of advertisement to successful recruitment. Such changes are not without possible benefits as well; casting a larger net that does not rely on social media platforms to assign people to interest group categories may lead to more inclusive samples in the future.

Our study is not without limitations. Because the primary goal of this project was not to understand engagement in various recruitment media, we do not have data describing the impact of web-based recruitment on continued study engagement of adolescents compared with other methods (eg, venue-based recruitment). It is possible that some adolescents who declined longitudinal participation may have done so because of the impersonal nature of the web-based recruitment approach. Furthermore, the use of social media apps by adolescents changes regularly. Although many youths in our study indicated that they still use Facebook or Instagram, the landscape of social media is constantly shifting, and researchers will need to be mindful of this or face mounting costs as users leave platforms that have fallen out of favor.

### Conclusions

Despite these limitations, key advantages of recruiting a sample using this approach warrant its ongoing use. First, we could target, screen, and carefully recruit a national sample within a relatively short period. Second, given the demographic differences in the cost to recruit people (eg, by age, gender, urbanicity, and sex assigned at birth), we rebudgeted to ensure successful recruitment in real time, adding and removing advertisements from the platforms in moments. This level of control proved useful, particularly because we recognized a need to expand our rural sample to address the sexual minority adolescent experience, as it applies to a more unique group that may not have the resources of those in urban settings. Finally, although the initial cost to recruit a national sample of diverse adolescents was more expensive than anticipated, the cost to retain them was similar to the nominal cost. Given the concerns regarding the ability to retain adolescents in studies over time (in general), particularly those with no direct in-person contact to build rapport, this was a welcome finding that we believe can support future studies with this and other vulnerable populations.

## Abbreviations

LGBTQ: lesbian, gay, bisexual, transgender, and queer
OR: odds ratio
RDS: respondent-driven sampling
SMASI: Sexual Minority Adolescent Stress Inventory
